# Corneal Subbasal Nerve Plexus Changes in Severe Diabetic Charcot Foot Deformity: A Pilot Study in Search for a DNOAP Biomarker

**DOI:** 10.1155/2018/5910639

**Published:** 2018-11-04

**Authors:** Anica Herlyn, Ruby Kala Prakasam, Sabine Peschel, Stephan Allgeier, Bernd Köhler, Karsten Winter, Rudolf F. Guthoff, Thomas Mittlmeier, Oliver Stachs

**Affiliations:** ^1^Department of Trauma, Hand and Reconstructive Surgery, Rostock University Medical Center, Schillingallee 35, 18057 Rostock, Germany; ^2^Department of Ophthalmology, Rostock University Medical Center, Doberaner Straße 140, 18057 Rostock, Germany; ^3^Institute for Automation and Applied Informatics, Karlsruhe Institute of Technology (KIT), Hermann-von-Helmholtz-Platz 1, 76344 Eggenstein-Leopoldshafen, Germany; ^4^Institute of Anatomy, University of Leipzig, Liebigstrasse 13, 04103 Leipzig, Germany

## Abstract

**Introduction:**

Diabetic neuroosteoarthropathy (DNOAP) early symptoms are unspecific, mimicking general infectious symptoms and rendering a diagnosis challenging. Consequently, unfavourable outcomes occur frequently, with recurrent foot ulceration, infectious complications, and eventually amputation. Corneal confocal microscopy (CCM) of the subbasal nerve plexus (SNP) is used to detect early peripheral neuropathy in diabetic patients without diabetic retinopathy. This pilot study was designed to determine if specific SNP changes manifest in severe DNOAP in comparison to a healthy control group.

**Methods:**

This pilot study utilized a matched-pair analysis to investigate SNP changes by in vivo CCM for 26 patients (mean patient age 63.7 years, range 27 to 78) with severe DNOAP defined by condition after the need for reconstructive foot surgery (*n* = 13) and a healthy control group (*n* = 13). Corneal nerve fibre length (CNFL), nerve fibre density (CNFD), nerve branch density (CNBD), average weighted corneal nerve fibre thickness (CNFTh), nerve connecting points (CNCP), and average weighted corneal nerve fibre tortuosity (CNFTo) were assessed as well as the general clinical status, diabetic status, and ophthalmologic basic criteria.

**Results:**

In vivo CCM revealed significantly reduced SNP parameters in the DNOAP group for CNFL (*p* = 0.010), CNFD (*p* = 0.037), CNBD (*p* = 0.049), and CNCP (*p* = 0.012) when compared to the healthy control group. Six patients (46%) of the DNOAP group suffered from diabetic retinopathy and none of the control group.

**Conclusions:**

This pilot study revealed a rarefication of SNP in all measured parameters in patients with severe DNOAP. We see a potential value of CCM providing a SNP-based biomarker for early stages of DNOAP prior to the development of any foot deformities that needs to be evaluated in further studies. This trial is registered with German Clinical Trials Register (DKRS) DRKS00007537.

## 1. Introduction

Diabetic neuroosteoarthropathy (DNOAP) of the foot is a rare but devastating complication, characterized by a sterile destruction of bones and joints accompanied by a diabetic peripheral neuropathy that is estimated to affect 0.8%–8% of the diabetic population [1]. The disease progression may lead to a loss of osteoligamentous architecture with loss of the plantigrade foot alignment [2, 3]. This progressive character of instability is associated with a serious impairment of the quality of life and an estimated chance of up to 49% to develop a recurrent ulceration with high risk for further complications as infections and eventually the need for consecutive amputation [2, 3]. DNOAP patients with present ulcerations have a 12 times higher risk of amputation, compared to those with ulcer-free feet [4]. The correct treatment of DNOAP patients is still a matter of controversy. As the current estimated annual limb amputation rate is 2.7%, the main objective of any therapy is not only to lower the occurrence of amputation [5] but also to provide the ability to sustain a chronically infection- and ulcer-free foot to maintain long-term walking independence [2, 6–8]. This can be reached in approximately 60% of patients with plantigrade foot alignment by applying nonoperative treatment with total contact casting [2]. Anyhow, up to 40% of patients require recasting, secondary minor surgery, or are placed on prolonged disability [2, 6]. In severe DNOAP with loss of plantigrade foot alignment and imminent complications (e.g., ulceration), corrective arthrodesis techniques with resection of the deformity and reconstruction arthrodesis are often necessary [9–12].

Since early symptoms are unspecific, mimicking general infectious symptoms in combination with reduced sensibility due to polyneuropathy in diabetic patients, diagnosis of DNOAP is challenging, with early stages frequently being misdiagnosed as diabetic foot, infection, or oedema. Expert consultation yielding a correct diagnosis is often delayed, resulting in advanced disease, lost plantigrade foot alignment, and imminent complications. Until now, no diagnostic tool or biomarkers identifying specific early symptoms have been available to allow early diagnosis before onset of structural changes [9]. Therefore, there is an immediate need for a noninvasive and reliable diagnostic approach to target peripheral nerve damage primarily responsible for the development of DNOAP in diabetic patients.

Previous ophthalmologic studies demonstrated a complex relationship between the subbasal nerve plexus (SNP), decreased corneal sensation, and the development of diabetic retinopathy and neuropathy [13]. Currently, SNP analysis is used as biomarkers in detecting early peripheral neuropathy in diabetic patients without diabetic retinopathy. In vivo corneal confocal laser scanning microscopy (CCM), a noninvasive corneal imaging technique, is frequently used to analyse cellular structures of the layers of the cornea, including small nerve fibres that compose the corneal SNP. Further, CCM had successfully demonstrated morphological changes of the SNP in corneal nerve fibre length, density, and diameter in patients with diabetes and those with impaired glucose tolerance directly associated with the progression of diabetic retinopathy [14]. The neurotraumatic theory [15] for pathogenesis of DNOAP is one of the two main theories that attempts to explain the development of DNOAP. This theory hypothesizes acute, subacute, or repetitive trauma as a causative factor in the setting of absent protective sensation, with the surrounding tissue responding with an acute-phase proinflammatory reaction of cytokines (tumor necrosis factor-*α*, interleukin-1*β*, and interleukin-6) [16]. While this theory does not explain all the changes seen in DNOAP, neuropathy does seem to play a major role in its development. Therefore, the aim of this pilot study is to determine if specific SNP changes manifest in severe DNOAP. Those might qualify as a biomarker to diagnose the disease at early stages prior to the development of any foot deformities.

## 2. Methods

Participants (*n* = 26) for this clinical study include 13 randomly chosen diabetic patients with severe DNOAP that were age- and gender-matched with 13 healthy participants (control group). Severe DNOAP was defined with the occurrence of bony deformities leading to a loss of the plantigrade foot alignment, with subsequent surgical corrective osteotomy with reconstructive arthrodesis. CCM reconstruction data were not available due to artefacts in two of the diabetic patients. A detailed interview, review of patients' medical records, and clinical investigation in the Department of Trauma, Hand and Reconstructive Surgery and Ophthalmological Department were conducted in January 2013 and August 2014.

The study was conducted in strict accordance with the Declaration of Helsinki and approved by the local Ethics Committee of the Medical Faculty of the University of Rostock (permit no. A 2012-0118). All participants were informed prior the investigation by telephone and face-to-face, and written consent was given.

### 2.1. Diabetical and General Clinical Status

Mean age of all participants (19 males, 7 females) was 63.7 ± 11.6 years (range 27–78) and was close to total mean between groups (Charcot 61.1 ± 13.6 years (range 27–77), control 66.4 ± 8.8 years (range 51–78)). Medical condition of all patients was assessed by review of patient-reported medical history and review of patients' charts. Clinical history of diabetes and Charcot disease was recorded by secondary diagnosis, glycated haemoglobin (HbA1c) levels, and basic patient characteristics. Neuropathology was analysed by monofilament testing adding to tuning fork and reflex hammer examination.

### 2.2. CCM and Ophthalmological Status

All subjects completed a full ophthalmological evaluation by a single ophthalmologist which includes a slit lamp examination and funduscopy. Corneal sensation measurements were obtained by performing Cochet-Bonnet esthesiometry. CCM was performed on unilateral eyes of all subjects. The used CCM technique was developed by combining a Heidelberg retina tomograph II (HRT II) with the Rostock cornea module (RCM) (Heidelberg Engineering GmbH, Heidelberg, Germany), as previously described [17]. The HRTII/RCM is equipped with immersion objective (Achroplan 63 × 0.95 W; Carl Zeiss Jena, Jena, Germany). For image acquisition, a disposable cap made of PMMA (TomoCap, Heidelberg Engineering GmbH) filled with a small amount of the contact gel Vidisic (Dr. Gerhard Mann Chem.-Pharm. Fabrik GmbH, Berlin, Germany) is put on the RCM lens. The Vidisic gel is also used as a coupling medium between the cornea and the TomoCap. Before image acquisition, the examined eye was anesthetized with Proparakain 0.5% eye drops (URSAPHARM, Saarbrücken, Germany). Using a modified version of the HRT II software (oscillating volume scan, operating mode), image stacks with an axial image distance of 0.5 *μ*m were acquired for each patient, with each stack representing a partial volume of the patient's cornea, including the SNP. At least three scans were performed for each patient, with a total duration of the image acquisition process of about 15 minutes. A specially developed image processing software allows the detection and correction of motion artefacts and ridge-like deformations. The result is a motion-corrected image of the subbasal nerve plexus reconstructed from each depth scan [18]. To create an expanded field of view, a mosaic image is generated by all reconstructed SNP images with common overlapping areas. Nerve structures and similar image features in the mosaic images were subsequently segmented. Incorrectly segmented structures (e.g., reconstruction artefacts, dendritic cells, and fibrotic tissue) were removed based on their morphological properties (size, elongation). In addition to the resulting image of the segmented nerve fibres, the network of fibre centrelines was finally calculated by thinning all segmented nerve fibres to a width of one pixel. Both the segmentation image and the thinned fibre network image formed the basis for the automated quantitative morphological and topological assessment of the SNP. The following CCM parameters were determined: corneal nerve fibre length (CNFL), defined as the total length of all nerve fibres (mm/mm^2^); corneal nerve fibre density (CNFD), defined as the number of nerve fibres per mm^2^; corneal nerve branch density (CNBD), defined as the number of branches per mm^2^; average weighted corneal nerve fibre thickness (CNFTh), measured as mean thickness perpendicular to the nerve fibre course (*μ*m); corneal nerve connecting points (CNCP), defined as the number of nerve fibres crossing area boundary (connections/mm); and average weighted corneal nerve fibre tortuosity (CNFTo), reflecting variability of nerve fibre directions and defined as total absolute nerve fibre curvature. Each fibre segment terminated by branching points, end points, and/or image borders was considered a distinct nerve fibre for the calculation of the above parameters. Weighting of single fibres was based on their contribution to the length of the total fibre network; for more details about the imaging and analysis process, see Köhler et al. [19].

### 2.3. Statistical Analysis

Age and gender were determined to be the most important factors for matching participants. All data were processed in Excel 2010 (Microsoft Corporation, Redmond, USA) and SPSS software package version 21.0 (SPSS, Chicago IL) and were used for statistical testing. Descriptive statistics were computed for continuous and categorical variables and are displayed as mean and standard deviations (SD). Kolmogorov-Smirnov test determined normality of the tested variables, and either independent *t*-test analyses or Mann-Whitney *U* test (nonnormal distribution) was performed to analyse the differences in outcomes between the two groups, with a significance level defined at *p* = 0.05. The effect size was calculated according to Cohens *d* and categorized into small (*d* = 0.2–0.5), medium (*d* = 0.5–0.8), and large (*d* > 0.8) effect sizes.

## 3. Results

### 3.1. Diabetical and General Clinical Status

All but 2 patients in the DNOAP group suffered from type 2 diabetes (*n* = 11); the remaining patients were diagnosed with type 1 diabetes (*n* = 2). Insulin dependency was manifest in 85% of patients. The mean duration since onset of the diagnosis in type 2 diabetes patients was 18.5 ± 9.7 years. Six patients (46%) suffered from 3 or more secondary diseases. All patients suffered from diabetes-associated polyneuropathy; 4 patients from a chronic renal insufficiency. Please see the detailed basic patient characteristics in Table 1.

None of the patients in the control group had diabetes or problems related to diabetes nor were any suffering from more than 3 secondary diagnoses. One patient with chronic urinary retention also suffered from chronic renal insufficiency, which likely developed after a stroke. None of the control patients had severe foot deformities, and all were in a healthy condition.

HbA1c levels in the DNOAP group were 8.2 ± 1.9% compared to 5.4 ± 0.5% in the control group.

### 3.2. DNOAP

**Figure 1:**
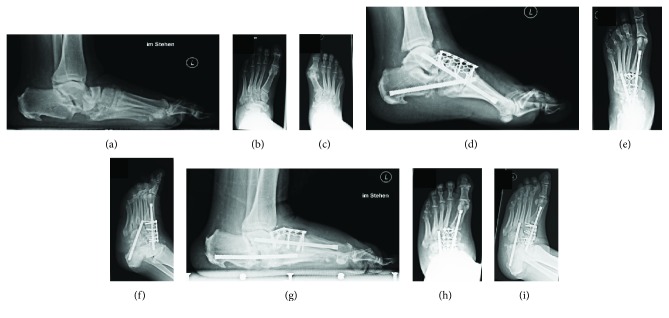
Radiologic case series of a 56-year-old male patient with IDDM for 19 years and severe midfoot Charcot arthropathy-type Sanders/Frykberg II/III on the left foot, with subluxation in the talocalcaneal and talonavicular joints. Initial diagnosis made in October 2013 after trivial distortion of the upper ankle joint (a–c) bearing radiographs in lateral, anterior-posterior, and oblique views. Intra- and extramedullary implants were used for reconstructive arthrodesis of the medial and lateral midfoot columns (d–f). Radiographs 6 months after surgery reveal a loss of reduction and implant breakage, displaying a frequent problem in Charcot surgery (g–i).

All patients in the DNOAP group were diagnosed with severe nonplantigrade Charcot foot deformity and had undergone surgical corrective osteotomy with reconstructive arthrodesis between November 2002 and November 2014 (6 right feet and 7 left feet). Applying the topographic classification of Sanders and Frykberg, a midfoot affection type II and/or III, corresponding to the Lisfranc and Chopart joint region, was causal in all but one foot. Further, per Sanders/Fryberg type IV, an involvement of the subtalar and talocalcaneal joints was visible in radiographs in 1 foot solely and 2 feet additionally. All feet were operated during the consolidation phase of Eichenholtz stage III. Surgical stabilization of the medial column was performed using extramedullary implants (*n* = 11, 85%; thereof *n* = 7, 53% angular stable plates) or intramedullary implants (*n* = 1, 8%) or a combination of both (*n* = 1, 8%). Stabilization of the lateral column was performed using extramedullary implants in 11 feet (85%). Additionally, hindfoot arthrodesis was performed in 1 foot (8%) via Ilizarov fixateur externe. A representative case is shown in Figure 1.

DNOAP initial diagnosis was at mean 0.7 ± 0.9 years (range 0–2) prior to reconstructive surgery. All but one DNOAP patient had ulcer-free, stable feet at the time of ophthalmological evaluation. In one case, a secondary amputation of the lower leg was performed due to uncontrollable infectious complications.

### 3.3. CCM and Ophthalmological Status

Six patients (46%) of the DNOAP group were diagnosed with diabetic retinopathy, 4 patients (31%) had received prior ocular treatment (all pseudophakia), 2 patients (15%) were diagnosed with glaucoma, and 3 patients (23%) were diagnosed with cataracts, compared to none of these conditions in the control group. There were no significant differences of corneal sensation between the two groups.

**Figure 2:**
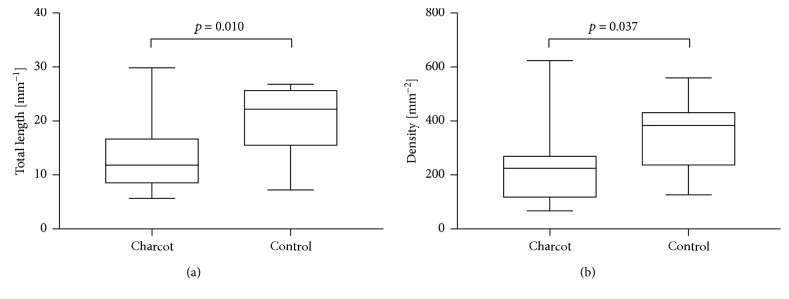
Corneal nerve fibre length defined as the total length of all nerve fibres (mm/mm^2^, *p* = 0.010) (a) and corneal nerve fibre density defined as the number of nerve fibres per mm^2^ (*p* = 0.037) (b) in Charcot and control groups.

**Figure 3:**
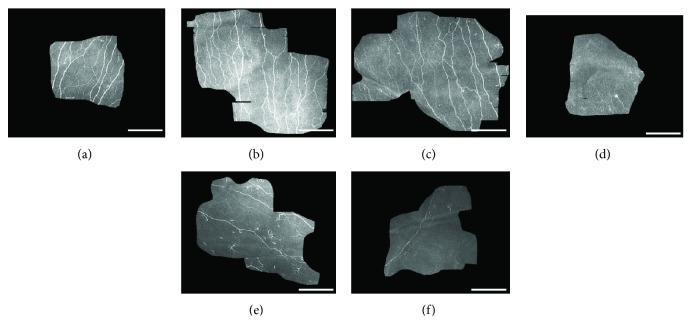
Confocal images demonstrating the morphology of SBN from 3 control subjects (a, b, c) and from 3 Charcot subjects (d, e, f). (d, e, f) Exhibited readily visible changes with decrease in nerve fibers, decrease in nerve branches and connectivity, and abnormal presence of widely scattered dendritic cells (e). Each scale bar represents 200 *μ*m.

In vivo CCM revealed significant changes in SNP morphology between the two groups (mean reconstructed SNP area: control 0.25 ± 0.06 mm^2^ (range 0.16–0.37), Charcot 0.19 ± 0.05 mm^2^ (range 0.13–0.28)). Corneal nerve fibre length (Charcot 13.08 mm/mm^2^, control 20.03 mm/mm^2^, *p* = 0.010, *d* = 1.09) and corneal nerve fibre density (Charcot 224.51/mm^2^, control 342.16/mm^2^, *p* = 0.037, *d* = 0.87) were significantly reduced for the DNOAP group as displayed in Figure 2. Representative confocal images from both study groups demonstrate readily visible SNP changes in Figure 3.

**Figure 4:**
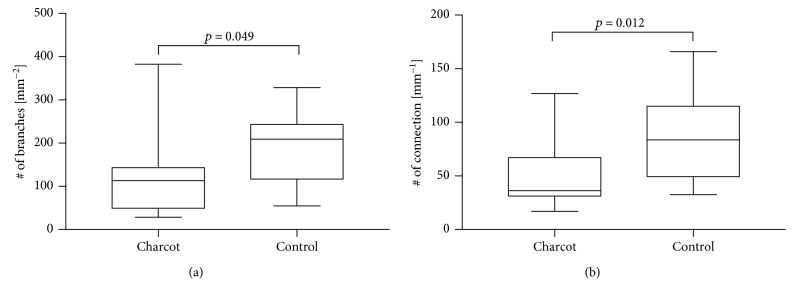
Corneal nerve branch density defined as the number of branches per mm^2^ (*p* = 0.049) (a) and corneal nerve connecting points defined as the number of nerve fibres crossing area boundary (*p* = 0.012) (b) in Charcot and control groups.

The Charcot group showed significantly reduced corneal nerve branch density (117.16/mm^2^) compared to the control group (187.45/mm^2^ in the control group (*p* = 0.049, *d* = 0.81)). Analogously, the corneal nerve connecting points (connections/mm) were highly significant with 49.75 connections/mm^2^ in the Charcot group compared to 88.11 connections/mm^2^ in the control group (*p* = 0.012, *d* = 1.06) (see Figure 4 for details).  Table 2 shows values for average weighted corneal nerve fibre thickness and average weighted corneal nerve fibre tortuosity. There were no significant relationships between reported changes in CCM findings and length of DNOAP or diabetic disease.

## 4. Discussion

Extensive research has focused on discovering the underlying pathology responsible for DNOAP-specific acute, uncontrolled, localized inflammation that may lead to the development of skeletal deformities that progress to the severe rocker-bottom foot with a collapsed plantar arch. The onset and the clinical presentation of DNOAP can mimic other common pathologies and is therefore often misdiagnosed or diagnosed at a later stage of the disease, causing inferior patient outcome [20, 21]. Therefore, it is essential to differentiate those patients with a higher risk of developing DNOAP from those who are not by using reliable methods of neurological examinations. The most commonly used neurological tools are capable of assessing large fibre neuropathy; however, both large (A-fibres) and small (C-fibres) fibre neuropathies are purported as possible causes of DNOAP [22]. Therefore, there is indication to study the density and morphological changes of small fibres, which provide mostly sensory and autonomic innervations, and to evaluate the possibility of early detection of DNOAP.

The cornea plays a pivotal role in the regulation of corneal sensation, maintenance of epithelial integrity, proliferation, and promotion of wound healing and is the most densely innervated tissue in the human body, supplied by terminal branches of the ophthalmic division of trigeminal nerve. Furthermore, it has been well established that the quantity and morphology of the corneal nerve fibres can reflect the status of diabetic polyneuropathy; therefore, the assessment of SNP serves as a novel ophthalmic marker for the detection of diabetic polyneuropathy [23, 24]. In vivo CCM, a noninvasive corneal imaging technique, is a useful and highly sensitive technique in the detection and assessment of systemic diseases with peripheral neuropathy, such as diabetes or polyneuropathic conditions. Increasing work on quantifying diabetic neuropathy using CCM has demonstrated that a significant reduction in corneal subbasal nerve fibre density and an increase in nerve fibre tortuosity in diabetes are correlated with the stage or severity of peripheral neuropathy [21–23]. In addition, CCM allows for the detection of early peripheral neuropathy, as the corneal nerve fibre damage precedes the development of diabetic retinopathy and impairment of corneal sensitivity [14]. As DNOAP is generally one of the late complications of diabetes and is primarily associated with diabetic polyneuropathy, assessment of corneal subbasal nerves might provide useful insight into the onset and progression of this condition.

While we are seeing an increase in the literature examining the pathophysiology of DNOAP, the work relating SNP changes to the DNOAP remains limited. One such study by Zhivov et al. [25] correlated diabetic foot syndrome and corneal subbasal nerve plexus changes and demonstrated decreased corneal sensation and decreased nerve fibre density in Congolese patients with type 2 diabetes with diabetic retinopathy and advanced foot ulceration; however, none of the patients suffered from DNOAP. The only CSLM study on DNOAP known by the authors is a longitudinal observational one-case study by Dehghani et al. [26] that demonstrated a rapid reduction of corneal nerve parameters prior to the development of foot ulcerations and DNOAP. While conventional measures of neuropathy did not deteriorate significantly on this type 2 diabetes patient, CSLM showed moderate to severe neuropathy at baseline followed by rapid decline in corneal nerve fibre density, nerve branch density, and fibre length over the duration of 7 years [26]. During this period, the patient developed a foot ulcer and DNOAP with the need for partial amputation. Similarly, our study demonstrated a significant reduction in corneal nerve fibre length, density, thickness, tortuosity, branch density, and connecting points of SNP in the DNOAP group when compared to the healthy control group. We rate these results as promising in search for a DNOAP biomarker with the eye displaying an ideal noninvasive diagnostic method. Anyhow, limitations of the study are the solely survey of a collective with severe DNOAP stages and the control group out of only healthy patients. In future studies, the method will likely be applied in comparing SNP changes in DNOAP patients with less severe stages and to further subgroups from diabetic patients, e.g., with diabetic neuropathy without DNOAP. Additionally, long-term, longitudinal observations of SNP changes in diabetic patients to discern specific changes prior to the onset of DNOAP are of interest.

## 5. Conclusions

This pilot study revealed significant changes with large effect size in all measured parameters of SNP for DNOAP when compared to healthy controls with basically rarefication of SNP. We see a potential value of CCM as noninvasive measure for diagnostics of DNOAP by qualifying SNP changes. Further studies are needed to evaluate if SNP changes can provide a biomarker to diagnose the disease at the early stages, prior to the development of any foot deformities.

## Figures and Tables

**Table 1:** Demographic data of included participants.

	Charcot	Control
Age	61.1 ± 13.6 years (range 27–77)	66.4 ± 8.8 years (range 51–78)
Gender	Male *n* = 10 (77%)	Male *n* = 10 (77%)
	Female *n* = 3 (23%)	Female *n* = 3 (23%)
Diabetes type	Type 2 *n* = 11 (85%)	None
	Type 1 *n* = 2 (15%)	
Diabetes duration	18.5 ± 9.7 years (range 1–31)	—
Insulin	Dependent *n* = 11	None
	Independent *n* = 2	
Secondary diagnosis (>3)	*n* = 6 (46%)	None
Polyneuropathy	*n* = 13 (100%)	None
Chronic renal insufficiency	*n* = 4 (31%)	*n* = 1 (8%)
Diabetic retinopathy	*n* = 6 (46%)	None
HbA1c levels	8.2 ± 1.9% (range 6–12)	5.4 ± 0.5% (range 5-6)

**Table 2:** Average weighted corneal nerve fibre thickness (CNFTh), measured as mean thickness perpendicular to the nerve fibre course (*μ*m) and average weighted corneal nerve fibre tortuosity (CNFTo), reflecting variability of nerve fibre directions, defined as total absolute nerve fibre curvature in both groups.

	CNFTh (mean value, in *μ*m)		CNFTo (mean value)	
	Charcot	Control	Charcot	Control
	2.25	2.31	1.30	2.07
	2.19	2.41	1.32	1.29
	2.27	2.25	1.50	1.49
	2.27	2.20	2.51	1.45
	2.28	2.23	1.82	1.19
	2.34	2.18	1.54	1.60
	2.21	2.33	1.20	3.00
	2.29	2.38	1.41	1.33
	2.30	2.32	1.33	1.36
	2.28	2.32	1.43	1.66
	2.20	2.28	1.25	1.33
	2.20	2.35	1.94	1.32
	2.16	2.27	1.96	1.61
∑ mean	**2.25**	**2.30**	**1.58**	**1.60**
SDV	**0.05**	**0.07**	**0.38**	**0.48**
Min	**2.16**	**2.18**	**1.20**	**1.19**
Max	**2.34**	**2.41**	**2.51**	**3.00**

## Data Availability

The data used to support the findings of this study are available from the corresponding author upon request.
